# Editorial: Physical neuromorphic computing and its industrial applications

**DOI:** 10.3389/fninf.2023.1238168

**Published:** 2023-07-17

**Authors:** Toshiyuki Yamane, Akira Hirose, Bert Jan Offrein

**Affiliations:** ^1^IBM Research - Tokyo, Tokyo, Japan; ^2^The University of Tokyo, Tokyo, Japan; ^3^IBM Research—Zurich, Rüschlikon, Switzerland

**Keywords:** analog neuromorphic devices, electric neuromorphic computing, material neuromorphic computing, quantum neuromorphic computing, IoT and edge computing

## 1. Introduction

The importance of handling cognitive data such as images, voices, and natural languages is wide spreading not only at datacenters but also at networking, edge, and IoT. Artificial neural networks are a powerful and prospective concept to process such cognitive data.

The improved performance of neural networks is achieved by increasing the scale of neural network models. This unavoidably has a direct and tremendous impact on the energy required to training and inference by current software and general-purpose processors due to their serial operation. On the other hand, current hardware acceleration is based on well-matured ASIC technology and integrated electronics. However, with the downscaling limit of conventional technologies, the traditional electronic computing will face difficulties in further growing in terms of energy efficiency.

To address the power and performance constraints, neuromorphic computing is a promising approach. In fact, various CMOS-based neuromorphic devices have been reported so far. In recent years, motivated by potential computational capabilities of various natural physical phenomena, unconventional computing paradigms have been actively investigated in the interdisciplinary region of computer science and natural science. The objective of this Research Topic is to investigate the possibility of incorporating diverse natural physical phenomena to neuromorphic computing, which we call “physical neuromorphic computing.”

In this Research Topic, we collected nine papers relevant to the theory, algorithm, and implementation of physical neuromorphic computing. They can be roughly classified into the following categories: electric, material, and quantum neuromorphic computing.

## 2. Electric neuromorphic computing

Undoubtedly, electric neuromorphic computing is the most actively investigated research area in neuromorphic computing, where non von-Neumann architectures are pursued with brain-like features such as distributed and sparse information representations, massive parallelism, event-driven operation, analog signal processing and on-chip learning capability.

Stapmanns et al. derived two efficient algorithms for archiving postsynaptic membrane potentials, based on event-based synapse updates and compared two algorithms with a time-driven synapse update scheme in terms of memory and computations. They showed that the two event-based algorithms significantly outperform the time-driven scheme. Their results on information archiving efficiency provide guidelines for the design of learning rules and make them practical in large-scale networks.

Michaelis et al. developed an open source emulator named Brian2Loihi for Loihi, which is a neuromorphic many core processor for spiking neural network with on-chip learning capability. They demonstrated error-free emulation for a single neuron and a recurrent spiking neural network and implementation of on-chip learning. Their work provides a quick prototyping and deployment of new algorithms for Loihi.

Hopkins et al. presented a new concept “sparse binary coincidence (SBC) memory” and its realization on surrounding infrastructure called BitBrain. The SBC memory stores coincidences between features in a training set and infers the class of a test example by identifying the class with which it shares the highest number of feature coincidences. They applied these concepts to the MNIST and EMNIST benchmarks and showed very low training costs and robustness to noise. BitBrain is designed to be implemented efficiently on both neuromorphic devices such as SpiNNaker and conventional CPU and memory architectures and is well-suited for edge and IoT applications.

Md Abdullah-Al Kaiser et al. proposed an asynchronous non von-Neumann analog processing-in-pixel architecture to perform convolutional multiply and accumulate (MAC) operations by integrating *in-situ* multi-bit multi-channel convolution inside the pixel array. They verified the architecture on vision sensor datasets and showed that the solution consumes significantly less energy than their digital MAC alternative; less than half of the backend-processor energy while retaining front-end energy and a high test accuracy.

## 3. Material neuromorphic computing

The major energy consumer of today's digital processor is data movement between MAC processors and volatile main memories. This motivates integration of memory and computation called “in-memory computing.” Use of intrinsic properties of materials for in-memory computing is becoming very promising approach for in-memory computing.

Gokmen and Haensch presented a new training algorithm, called “Tiki-Taka” algorithm for deep neural networks on resistive cross-point device arrays. Tiki-Taka alleviates stringent symmetry requirement that resistive devices must change conductance symmetrically for positive or negative pulse stimuli. Simulation results show that the accuracy of the SGD algorithm with symmetric device switching characteristics is matched in that of the Tiki-Taka algorithm with non-symmetric device switching characteristics.

Corti et al. presented an in-memory computing platform for convolutional neural networks by synchronization in phase and frequency of coupled VO2 oscillators. The neuromorphic architecture was fabricated in a crossbar configuration on silicon and achieved significant improvements of area density, oscillation frequency, variability and reliability, compared to existing digital convolutional filters. They applied the platform to MNIST recognition task and achieved high recognition accuracy.

Garg et al. demonstrated that the phase synchronization of glial cells can be reproduced by injected radio-frequency signals in the heavy metal layer of spin-orbit torque oscillators. They also proposed applications of such neural synchronization to the temporal binding problem and the design of a coupled neuron-synapse-astrocyte network.

## 4. Quantum neuromorphic computing

Quantum computing is an emerging technology based on completely different principles from classical computers, the laws of quantum mechanics. Quantum computing, if it happens in reality, has the potential to solve many industrial problems that classical computers cannot with a reasonable amount of resources. The nonlinearity of the quantum devices used in quantum computing can be applied to energy-efficient neuromorphic computing.

Tschirhart and Segall investigated how superconducting electronics by Josephson junctions address the requirements for large scale neuromorphic systems, such as scalability, programmability, biological fidelity, on-line STDP learning, efficiency, and speed. The result of detailed numerical analysis based on digital logic demonstrations showed that superconducting electronics is suitable for fast and efficient neuromorphic experimental platform in the future.

Rahman et al. reported a differential device by Fowler-Nordheim (FN) quantum-mechanical tunneling. They showed that a prototype FN-synapse array can achieve near-optimal memory consolidation characteristics with tunable plasticity-stability trade-offs, compared to other physical implementations. They also claimed that the proposed FN-synapse provides an ultra-energy-efficient approach for implementing both synaptic memory consolidation and continual learning in terms of an energy footprint per synaptic update.

## Author contributions

All authors listed have made a substantial, direct, and intellectual contribution to the work and approved it for publication.

